# Beyond Chelation: EDTA Tightly Binds Taq DNA Polymerase, MutT and dUTPase and Directly Inhibits dNTPase Activity

**DOI:** 10.3390/biom9100621

**Published:** 2019-10-17

**Authors:** Anna Lopata, Balázs Jójárt, Éva V. Surányi, Enikő Takács, László Bezúr, Ibolya Leveles, Ábris Á. Bendes, Béla Viskolcz, Beáta G. Vértessy, Judit Tóth

**Affiliations:** 1 Institute of Enzymology, Research Centre for Natural Sciences, Hungarian Academy of Sciences, 1113 Budapest, Hungary; lopata.anna@gmail.com (A.L.); suranyi.eva@ttk.mta.hu (É.V.S.); enikot@gmail.com (E.T.); leveles.ibolya@ttk.mta.hu (I.L.); abris.bendes@oulu.fi (Á.Á.B.); vertessy@kutatok.org (B.G.V.); 2 Department of Applied Biotechnology, Budapest University of Technology and Economics, 1111 Budapest, Hungary; 3 Institute of Biophysical Chemistry, Goethe University, 60438 Frankfurt am Main, Germany; 4 Institute of Food Engineering, Faculty of Engineering, University of Szeged, 6724 Szeged, Hungary; jojartb@gmail.com; 5 Department of Inorganic and Analytical Chemistry, Budapest University of Technology and Economics, 1111 Budapest, Hungary; bezur@mail.bme.hu; 6 Faculty of Biochemistry and Molecular Medicine, University of Oulu, 90220 Oulu, Finland; 7 Institute of Chemistry, University of Miskolc, 3515 Miskolc, Hungary; bela.viskolcz@uni-miskolc.hu

**Keywords:** dNTP hydrolysis, EDTA, dNTP pool sanitizing enzymes, dNTPase inhibitor

## Abstract

EDTA is commonly used as an efficient chelator of metal ion enzyme cofactors. It is highly soluble, optically inactive and does not interfere with most chemicals used in standard buffers making EDTA a common choice to generate metal-free conditions for biochemical and biophysical investigations. However, the controversy in the literature on metal-free enzyme activities achieved using EDTA or by other means called our attention to a putative effect of EDTA beyond chelation. Here, we show that EDTA competes for the nucleotide binding site of the nucleotide hydrolase dUTPase by developing an interaction network within the active site similar to that of the substrate. To achieve these findings, we applied kinetics and molecular docking techniques using two different dUTPases. Furthermore, we directly measured the binding of EDTA to dUTPases and to two other dNTPases, the Taq polymerase and MutT using isothermal titration calorimetry. EDTA binding proved to be exothermic and mainly enthalpy driven with a submicromolar dissociation constant considerably lower than that of the enzyme:substrate or the Mg:EDTA complexes. Control proteins, including an ATPase, did not interact with EDTA. Our findings indicate that EDTA may act as a selective inhibitor against dNTP hydrolyzing enzymes and urge the rethinking of the utilization of EDTA in enzymatic experiments.

## 1. Introduction

Divalent metal ions including Mg^2+^, Cu^2+^, Fe^2+^, Mn^2+^, Ni^2+^, and Zn^2+^ play prominent roles in enzymatic catalysis. The small organic compound ethylene diamine tetraacetic acid or commonly known as EDTA is frequently used to chelate these metal ions to investigate enzyme function in the absence of metal co-factors. This molecule comprises four carboxylic acid groups, thus have an overall negative charge. Its structure is flexible when unbound and adopts a rigid conformation while complexed with a single divalent metal ion (Me^2+^). All four carboxylic acid groups and two amines are involved in the hexacoordinated EDTA:Me^2+^ complex. It is highly soluble, optically inactive, and does not interfere with most chemicals used in standard buffers. Thus, EDTA seems to be a beneficial and safe choice to generate metal-free conditions for biochemical and biophysical investigations.

For enzymes catalyzing the hydrolysis of nucleotides to yield (d)NDP and P_i_ (inorganic phosphate) (e.g., ATPases [1], GTPases [2]) or (d)NMP + PP_i_ (e.g., ligases [3], DNA and RNA polymerases [4], Nudix hydrolases [5], and dUTPases) metal ions, notably Mg^2+^, are usually considered to be of high functional importance. In most cases, k_cat_ (steady-state catalytic activity) values are reported to decrease by several orders of magnitude in lack of these metal ions. Nucleotide hydrolyzing enzymes can operate with either one or two Mg^2+^ ions bound. The enzyme dUTPase is a preventive DNA repair protein that hydrolyses dUTP into dUMP and pyrophosphate, performing a dNTP pool sanitizing role. Thereby it prevents uracil incorporation into DNA that would eventually lead to hyperactive DNA repair cycles and cell death [6]. In most species, it forms a homotrimer with three active sites that are built up from five conserved motifs [6]. The substrate dUTP-Mg^2+^ complex binds to the active site and is coordinated by multiple H-bonds and a π-π stacking interaction [7]. Numerous publications provided experimental data indicating that the lack of the Mg^2+^ co-factor decreases the k_cat_ of dUTPase only by a factor of two [8,9,10,11,12]. Some publications, however, concluded the total inactivity of dUTPase without Mg^2+^ [13,14,15]. In the latter cases, high concentration of EDTA was used, or the method of providing metal-free conditions was not described.

To investigate the underlying cause of this controversy, we studied the effect of EDTA on dUTP hydrolysis catalysed by two different dUTPases (from *Homo sapiens*, hDUT, UniProt ID: P33316); and from *Mycobacterium tuberculosis*, mtDUT, UniProt ID: P9WNS5) in Mg^2+^-free conditions. We observed kinetic inhibition by EDTA in the absence of Mg^2+^. We then measured direct binding of EDTA to dUTPases and 5 other enzymes (including another dNTP pool sanitizing enzyme [16,17] and a DNA polymerase) using isothermal titration calorimetry. Our results show that EDTA binds selectively to the nucleotide binding pocket of dNTP processing enzymes. To understand the structural basis of this specific binding, we took a computational approach and explored the interactions of EDTA within the active site of dUTPases. We found that EDTA develops similar interactions with the protein to those of the cognate substrate.

## 2. Materials and Methods 

### 2.1. Reagents

We used our previously cloned N-terminally His-tagged dUTPases of human (hDUT and A98F hDUT) and of *Mycobacterium tuberculosis* origin (mtDUT). The *Escherichia coli* MutT (EcMutT) plasmid was a kind gift of Umesh Varshney, Indian Institute of Science, Bangalore, India. These proteins were expressed and purified as described previously [17,18,19,20]. We also used TEMpase Hot Start DNA polymerase from VWR (Radnor, PA, USA), a modified *Thermus aquaticus* originating DNA polymerase (Taq polymerase). This protein was heat activated for 15 min at 95 °C before the ITC measurement. Furthermore, we used our previously cloned *N*-terminally His-tagged *Staphylococcus aureus* uracil glycosylase inhibitor (SaUGI) [21] and *Petroselinum crispum* phenylalanine ammonia-lyase (PcPAL) [22] proteins. The rabbit skeletal myosin subfragment-S1 was a kind gift of Máté Gyimesi, Eötvös University, Budapest, Hungary. These proteins were expressed and purified as described previously [23,24,25]. The proteins were dialyzed against a buffer pH 7.5 comprising 20 mM HEPES, 100 mM NaCl and 1 mM TCEP. Protein concentration was determined by UV absorbance (ε_280_ = 10430 M^−1^cm^−1^ for hDUT, ε_280_ = 15930 M^−1^cm^−1^ for A98F hDUT, ε_280_ = 2980 M^−1^cm^−1^ for mtDUT, ε_280_ = 28990 M^−1^cm^−1^ for EcMutT, ε_280_ = 112760 M^−1^cm^−1^ for Taq polymerase, ε_280_ = 21890 M^−1^cm^−1^ for SaUGI and ε_280_ = 45,840 M^−1^cm^−1^ for PcPAL) or using the Bradford assay and is given in monomers. Other reagents were from Sigma-Aldrich (St. Louis, MO, USA).

### 2.2. ICP-OES

The Mg content of the solutions used in our experiments was determined by inductively coupled plasma optical emission spectrometry. Instrument settings were Labtest Plasmalab ICP spectrometer with 40 element vacuum polychromator, wavelength: 279.553 nm, 27 MHz Ar-Ar plasma, forward power: 1.3 kW, sample introduction with V-groove nebulizer at 2.8 mL/min sample flow rate. Limit of quantitation for Mg was 0.001 mg/L (0.04 µM).

### 2.3. Photometric Enzyme Activity Measurement

Continuous pH indicator-based assays were performed to measure enzyme activity at 20 °C as described in [26]. Briefly, 1 µM protein was used in a buffer pH 7.5 containing 1 mM HEPES, 100 mM KCl and 40 µM phenol red. A Specord 200 (Analytic, Jena, Germany) spectrophotometer and 10-mm-path length cuvettes were used, and absorbance was recorded at 559 nm. Initial velocity was determined from the first 10% of the progress curve. To explore the inhibitory effect of EDTA on both enzymes, we added this compound to the reaction mixtures at 30 and 100 µM concentration for hDUT and at 300 µM concentration for mtDUT. Data fitting was accomplished using OriginPro 7.5 (OriginLab Corp, Northampton, MA, USA).

### 2.4. Thermofluor Stability Assay

Solutions of 40 µM (0.8 mg/mL) protein in 20 mM HEPES pH 7.5, 100 mM NaCl, 10 mM ß-ME also containing 1000x dilution of Sypro^®^ Orange supplemented either with 5 mM MgCl_2_ or 100 µM EDTA were added to the wells of a 96-well thin-wall ABgene^®^ PCR plate (Thermo Fisher Scientific, Waltham, MA, USA). Apo enzyme, dUPNPP-complexed and dUMP-complexed enzymes were tested with the addition of saturating concentration of the ligand (100 µM dUPNPP or 1 mM dUMP) where applicable. The plates were sealed with ABgene^®^ Adhesive PCR Plate Sealing Tape (Thermo Fisher Scientific, Waltham, MA, USA) and heated in a Mx3000Pro QPCR System (Agilent Technologies, Santa Clara, CA, USA) from 25 to 90 °C. Fluorescence changes in the wells of the plate were monitored simultaneously with a photomultiplier tube (PMT). The wavelengths for excitation and emission were 492 and 516 nm, respectively.

### 2.5. Protein and Ligand Preparation Procedure for Molecular Docking

The protonation states of the titratable groups were determined by means of the ProPka algorithm [27,28] at pH 7.4, and the optimization of H-bonding interactions was performed using the PDB2PQR program package version 2.1.1 [29]. The original dUPNPP ligand in both human and *Mycobacterium tuberculosis* dUTPase crystal structures (PDB IDs: 3EHW, under publication in a separate paper and 2PY4 [20], respectively) was manually modified to dUTP.

The structure of the ligand (both EDTA and EDTA-Mg^2+^ complex) was taken from that of 1ZLQ [30]. The Fe^3+^ ion was replaced by a Mg^2+^ ion, and the coordination complex with EDTA was prepared manually using the Molecular Operating Environment 2007.09 program package (Chemical Computing Group, Montreal, Canada) [31]. Subsequent minimization was performed using the MMFF94x force field [32,33,34,35,36]. Gasteiger charges [37] were assigned to the enzyme and to the ligand using the same software.

### 2.6. Molecular Docking

Docking calculations were performed by means of the AutoDock version 4.2.3 software [38] using default settings and parameters as follows. The protein was kept rigid in the calculations and the ligand was allowed to be flexible. Lennard-Jones parameters 12–10 and 12–6 were used for modelling H-bonds and van der Waals interactions, respectively. To calculate the electrostatic grid map, the distance-dependent dielectric constant of Mehler and Solmayer was utilized. The Lamarckian Genetic Algorithm [39] with the pseudo-Solis and Wets method was used in the docking procedure, with 300 individuals in the population. The stopping criterion was defined by setting the total number of energy evaluations to 2.0 × 10^7^. The translation step was set to 5 Å/step, and in both quaternion and torsion steps 5.0 degrees/step was applied. The docking procedure was performed 25 × 16 (400) times for each enzyme-ligand complex.

Both blind docking [40,41] and active site docking calculations were performed. In blind docking, the grid box was centered on the whole protein and the number of grid points was set to 176 × 144 × 176 for both hDUT and mtDUT. In active site docking, the grid box was centered on the geometric center of dUTP and was large enough to accommodate the dUTP. The number of grid points was set to 42 × 34 × 46 and 46 × 34 × 38 for hDUT and mtDUT, respectively. The lattice point distance was set to 0.375 Å for every docking calculation.

### 2.7. Determination of the Enzyme-Ligand Interaction Network

The ‘Ligand interactions’ module [42] of MOE 2007.09 (Chemical Computing Group, Montreal, Canada) [31] was used for the 2D depiction of enzyme ligand interactions. The algorithm to determine the interaction sites in the ‘Ligand interactions’ module includes the following considerations: (1) Only heavy atom distances are considered; (2) first those residues are determined as interaction sites which are in a 4.5 Å proximity of any ligand atoms; (3) thereafter the distance limit is increased by 0.1 Å, and a new residue is determined as possible site if at least 2 atoms of that residue are within the new distance criteria; (4) increasing the distance by 0.1 Å and the number of the interacting atom by 1, this step is repeated 10 times. The possible H-bond interactions are determined by applying a scoring function. The strength of the H-bond is expressed as percentage probability. We applied a 10% cut-off in identifying possible H-bond interactions. 

### 2.8. Isothermal Titration Calorimetry (ITC) Measurement 

ITC experiments were carried out at 293 K on a Microcal ITC_200_ instrument (Malvern Instruments, Malvern, UK), following previously described experimental design [43]. The proteins were dialysed against a buffer pH 7.5 comprising 20 mM HEPES, 100 mM NaCl and 1 mM TCEP. We used 10–250 µM enzyme (hDUT, A98F hDUT, mtDUT, EcMutT, Taq polymerase) in the cell and 70–500 µM EDTA or 500–1500 µM dUPNPP in the syringe. For SaUGI, PcPAL and rabbit myosin S1, 20–80 µM enzyme was used in the cell and 40–120 µM EDTA in the syringe. Next, 5 mM MgCl_2_ was used in the syringe to titrate 500 µM EDTA in the cell. Protein concentrations correspond to subunits. Enzymes were also titrated with buffer to consider mixing and dilution heat effects. The titrations were performed with the injection syringe rotating at 750× *rpm* (and at 200× *rpm* for mtDUT). A series of 20 injections spaced 180 s apart from each other was performed with injection volumes 0.5 µL for the first and 2 µL for the subsequent 19 injections. All measurements were carried out in duplicates.

The integration of the obtained isotherms was performed using NITPIC v1.2.5 [44] and the global analysis was performed using SEDPHAT v12p1b [45,46]. The fitting model in SEDPHAT was A + B to AB heteroassociation (1:1 binding model), thereby obtaining stoichiometry (*n*), apparent binding affinity (K_a_) and enthalpy change (ΔH) parameters. The fitting was followed by export to GUSSI v1.3.2 to plot the processed data for publication-quality figure preparation [47].

### 2.9. Figures

Kinetic and thermostability graphs were prepared using OriginPro 7.5 (OriginLab Corp., Northampton, MA, USA). ITC graphs were prepared by GUSSI v1.3.2 [47]. Ligand interaction network images were prepared by MOE 2007.09 [31]. Structural images were prepared using the Visual Molecular Dynamics package [48] or the PyMOL Molecular Graphics System, Version 2.0 Schrödinger, LLC (New York, NY, USA) [49].

## 3. Results

### 3.1. Assessment of the Mg^2+^ Content of the Assay Solutions

To be able to measure the specific effects of EDTA, we needed to establish Mg^2+^-free or low Mg^2+^ concentration conditions without the use of EDTA. Therefore, we used high purity reagents and measured the exact Mg content of our buffers with atomic spectroscopy. The high sensitivity inductively coupled plasma optical emission spectrometry yields a quantitation limit of 0.001 mg/L for any oxidation state of Mg. The Mg content of our dH_2_O, dialysis buffer and dUTP nucleotide stock fell under the quantitation limit. The concentration of other divalent or trivalent metals (Mn, Ni, Co, Cr, Sr) that could potentially complement the function of Mg^2+^ fell under the quantitation limit, as well. However, the Mg content of our activity assay buffer was measured to be 0.007 ± 0.00014 mg/L (0.3 µM) despite the fact that we purchased the finest chemicals available. The smallest reported dissociation constant for the ATP:Mg^2+^ complex is 46.3 µM [50]. We considered this value to calculate the equilibrium concentration of the dUTP:Mg^2+^ complex possibly present in our reaction mixtures. As we used a range of 2–90 µM dUTP in our kinetics measurements, the calculated concentrations of the dUTP:Mg^2+^ complex were 0.02–0.4 µM in the solutions, i.e., at most 1% of the total dUTP was complexed with Mg^2+^ in our assays.

### 3.2. EDTA Decreases Enzyme Activity

**Figure 1:**
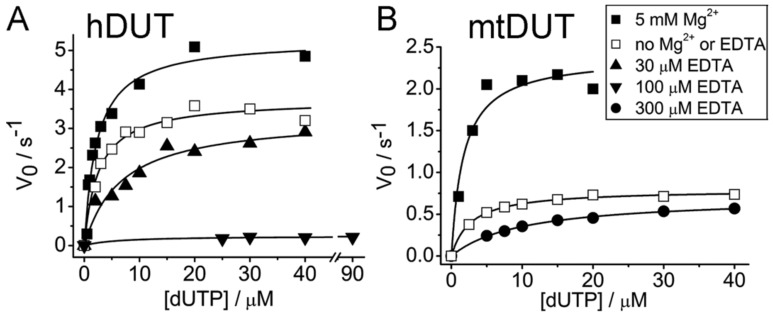
EDTA inhibits the enzyme activity of dUTPase. Figure shows Michaelis-Menten curves of hDUT (**A**) and mtDUT (**B**) in Mg^2+^-saturated condition (solid square), without Mg^2+^ or EDTA (open square) and in the presence of EDTA (solid triangles and circle). K_M_ and k_cat_ values of the fitted datasets can be found in Table 1.

We measured the dUTP hydrolysing activity of hDUT and mtDUT in the presence of Mg^2+^ (reported previously [20,51]) or EDTA and in the absence of both compounds (Figure 1, Table 1). In the absence of Mg^2+^, the activity decreased to 70% and 33%, while the K_M_ increased with 10% (within experimental uncertainty) and 50% for hDUT and mtDUT, respectively. The relatively modest change in the kinetic parameters upon lowering the Mg^2+^ concentration close to zero was not unexpected. We previously investigated the reaction mechanism of these enzymes using transient kinetics and QM/MM methods and found that the primary role of the Mg^2+^ in this system is to enforce a coordination constraint upon the enzyme–substrate complex which drives the reaction towards the transition state [51,52]. The rate-limiting proton-transfer reaction during hydrolysis is not directly dependent on the Mg^2+^ ion and therefore, a complete abolishment of the reaction is not expected in the absence of Mg^2+^ [52]. What was unexpected, however, is that the addition of EDTA further perturbed the enzyme kinetic parameters (Figure 1, Table 1). hDUT was inhibited even by as low as 30 µM EDTA so that the K_M_ increased 2.7-fold. 100 µM EDTA practically abolished the activity decreasing the k_cat_ to 6%. mtDUT was adversely affected by only higher concentrations of EDTA (300 µM) increasing the K_M_ 3.7-fold compared to that measured in the absence of Mg^2+^ and EDTA (Table 1).

The increase in K_M_ suggests competitive inhibition, meaning that EDTA competes with the ligand for the same binding site. Decreasing k_cat_ indicates non-competitive inhibition, i.e., the inhibitor binds to somewhere else other than the active site. Our results suggest that EDTA binds to the active site of both hDUT and mtDUT and it may additionally bind to other binding site(s), as well. The calculated 1–0.44% dUTP:Mg^2+^ content of the total dUTP present throughout the measured concentration range clearly does not account for the relatively high, 70% and 33% residual activity obtained for hDUT and mtDUT, respectively. Thus, these results strengthen the observations reported in [8,9,10,11] that Mg^2+^ or other divalent metal ions only increase, not enable, the hydrolytic activity of dUTPase roughly by a factor of two. 

### 3.3. EDTA Does Not Destabilize the Enzyme

**Figure 2:**
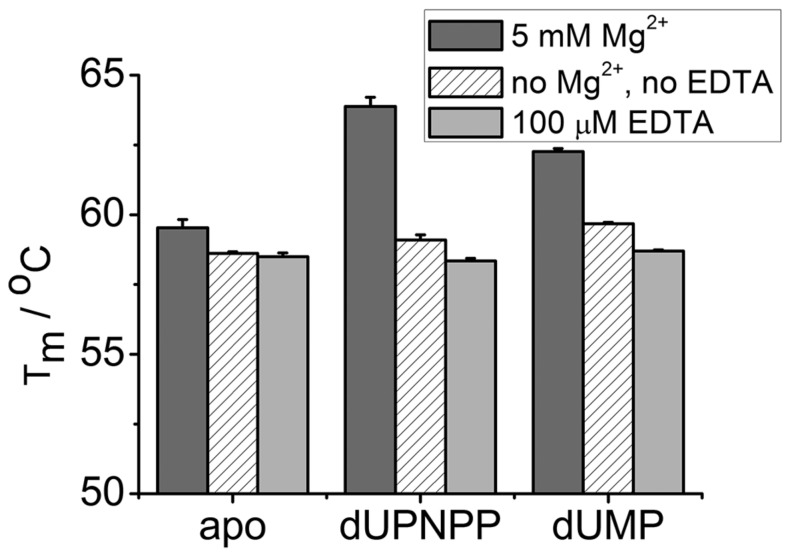
Melting temperatures of hDUT in the presence and absence of Mg^2+^ and EDTA. The apo enzyme and both complexes with the substrate analogue dUPNPP and the product dUMP were measured in Mg^2+^-saturated condition (dark gray), without Mg^2+^ or EDTA (white with stripes) and in the presence of EDTA (light gray). Calculated melting temperatures can be found in Table 2.

To exclude possible detrimental effects of EDTA on the structural stability of dUTPases, we measured the melting temperatures of hDUT in the presence of 5 mM MgCl_2_, of 100 µM EDTA or in the absence of both (Figure 2, Table 2). We chose hDUT as the steady state kinetic experiments showed that EDTA has a greater effect on hDUT than on mtDUT. The thermofluor data revealed that the thermostability of the enzyme is similar in the presence and absence of EDTA (Table 2). In the presence of Mg^2+^, the melting temperatures of the apo, dUMP and dUPNPP complexed enzymes increase with 0.9, 2.6 and 3.8 °C, respectively (Table 2). These results suggest that Mg^2+^ stabilizes the protein, especially when complexed with the substrate analogue or with the product. The results also suggest that EDTA does not have an effect on enzyme stability that could account for the largely decreased activity.

### 3.4. Blind Docking Indicates EDTA Binding to the Surface and the Active Site of Both dUTPases

**Figure 3:**
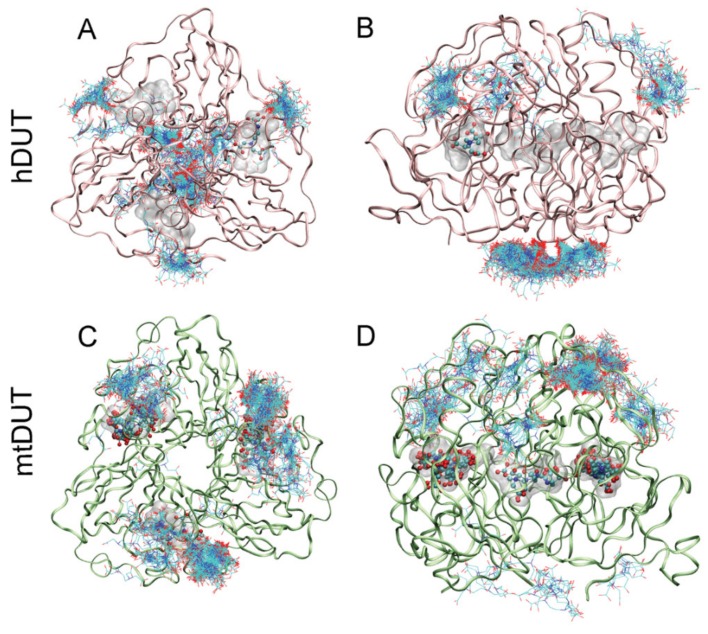
Blind docking of EDTA to dUTPase enzymes. Figures show all 400 docked EDTA molecules to hDUT (**A**,**B**) and mtDUT (**C**,**D**). Panels B and D show the same structure as A and C, respectively, upon rotating the protein by 90°. The active sites of dUTPases contain bound dUTP represented with the transparent space filling method (Maximal Speed Molecular Surfaces [53]). EDTA molecules bound to the active site are shown with ball and stick representation, whereas EDTA molecules bound to the surface of the enzyme are shown as only stick representation. Protein backbone is shown as pink and green ribbon for hDUT and mtDUT, respectively.

To identify potential binding sites of EDTA on the dUTPase enzyme, we chose to apply blind docking to both hDUT (PDB ID: 3EHW) and mtDUT (PDB ID: 2PY4 [20]) using the AutoDock 4.2.3 program package [38]. In these calculations, the whole protein structure is considered as potential binding surface, thus the ligand can explore distinct binding cavities and modes, as well. The blind docking calculations were performed 400 times for both EDTA and dUTP ligands. Among these runs, EDTA recognized the orthosteric binding site in hDUT and mtDUT 1 and 18 times, respectively. In the remaining cases, the surface of the protein was identified as EDTA recognition site (Figure 3). For comparison, the physiological substrate dUTP bound to the orthosteric cavity 5 and 22 times in hDUT and mtDUT, respectively. Thus, the obtained small numbers of orthosteric cavity exploration by EDTA could still represent effective binding to the active site. The difference in the number of active site bound ligands in hDUT and mtDUT might originate from the difference in their surface polarity. 16.3% and 10.1% of the protein surface is positively charged (arginine or lysine residues) in hDUT and in mtDUT, respectively. In effect, the larger basic surface in hDUT can result in more frequent binding on the surface of the protein by the negatively charged EDTA and dUTP. These results suggest that EDTA binds both to the surface and the active site of the dUTPase enzyme. 

### 3.5. Molecular Docking Suggests That Nucleotide and EDTA Share Common Interaction Points Within the Active Site

**Figure 4:**
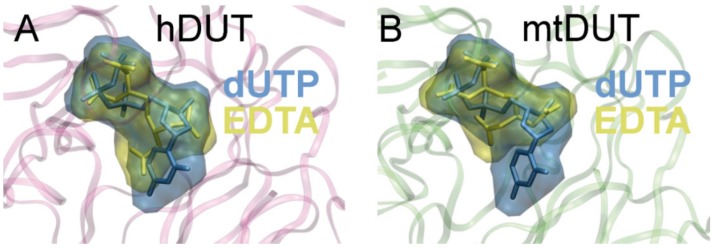
Molecular docking shows that EDTA occupies the same binding site in the dUTPase active site as the cognate ligand, dUTP. The Maximal Speed Molecular Surfaces [53] of dUTP (blue) and EDTA (yellow) largely overlap in both (**A**) hDUT and (**B**) mtDUT. Both ligands are also shown as stick representation besides the transparent surface representation. Protein backbone is shown as pink and green ribbon for hDUT and mtDUT, respectively.

To investigate the binding conformation of EDTA in the active site of dUTPase, we performed active site docking. For comparison, docking with the cognate ligand, dUTP, was also performed to the same protein structures. The accuracy and reliability of the docking simulation is confirmed by the low RMSD values (0.001 Å for both mtDUT and hDUT) comparing the docked dUTP to the dUPNPP within the crystal structure. The low RMSD value implies that the docking process reproduced the location and the conformation of the physiological ligand in the crystal structures. Figure 4 shows that the physiological ligand, dUTP, and EDTA explore the same space within the active site. In particular, EDTA overlaps with the phosphate chain and the sugar in both structures and in hDUT, EDTA also overlaps with the uracil ring of the physiological ligand. We also performed docking of the EDTA:Mg^2+^ complex to the dUTPase active sites and obtained ΔG_bind_ values close to zero. This implies that this complex is unlikely to productively bind to the active site and emphasizes the role of the free negative charges and/or the flexibility of EDTA in its interaction with the assayed proteins.

**Figure 5:**
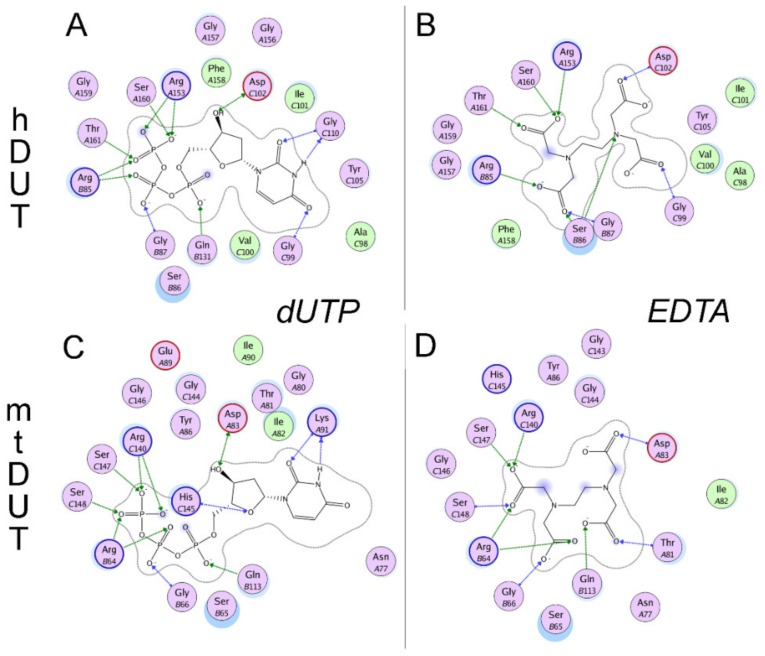
Comparison of the interaction networks between dUTPase:dUTP and dUTPase:EDTA complexes obtained by docking. (**A**) hDUT:dUTP complex, (**B**) hDUT:EDTA complex representing the most populated structure from active site docking, (**C**) mtDUT:dUTP complex, (**D**) mtDUT:EDTA complex representing the most populated structure from active site docking. Residues shown are within 5.4 Å from the ligand potentially interacting with it as determined by the ‘Ligand interactions’ algorithm [42] of MOE 2007.09 [31]. Color coding is as follows: Lilac background, polar residue; green background, apolar residue; blue border, basic residue; red border, acidic residue; blue halo, solvent accessible residues; blue smudge, solvent accessible atoms of the ligand; green arrow, H-bonds with residue side chain; blue arrow, H-bonds with residue backbone. No Mg^2+^ ions were present in this docking simulation. Ionic interactions between arginine sidechains and the phosphate chain of dUTP or the acetate part of EDTA were treated as H-bonds by the algorithm.

We also mapped the secondary interactions within the enzyme-EDTA complexes and compared this interaction network to that of the enzyme:dUTP complexes (Figure 5.). The H-bonding interactions in particular are compiled in Table 3. All of the residues predicted to interact with EDTA are found in one of the five conserved motifs present in all dUTPases [6]. Arg85/64 (amino acid numbering in hDUT and mtDUT, respectively), Asp102/83 and Arg153/140 are key and conserved amino acids in the binding site and were predicted as anchor points in EDTA binding, too. These residues are responsible for the coordination of the catalytic water (Asp102/83) and of the phosphate chain [54,55,56]. Two important residues in binding the γ-phosphate of the nucleotide substrate, Ser160/147 and Thr161/Ser148 [56,57] were determined to be key participants in the enzyme–EDTA interaction as well. These shared anchor points suggest that EDTA binds to the same site within the enzyme active site as the physiological substrate dUTP.

### 3.6. Isothermal Titration Calorimetry Data Directly Prove EDTA Binding to the Nucleotide Binding Pocket of dUTPase

**Figure 6:**
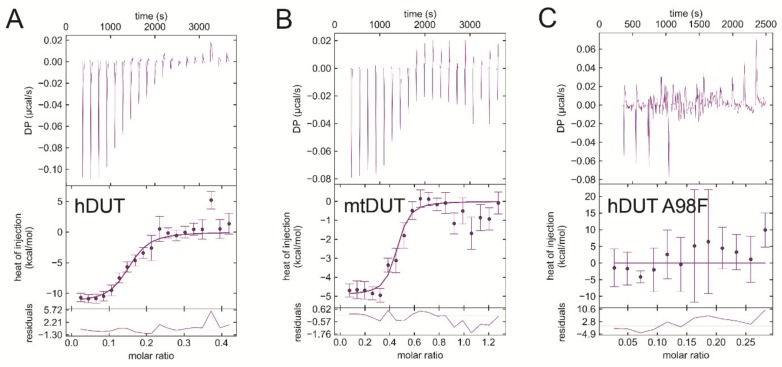
Isothermal titration calorimetry (ITC) titrations of wild-type and active-site mutant dUTPases with EDTA prove strong and direct EDTA binding to the active site. (**A**) hDUT titrated with EDTA. (**B**) mtDUT titrated with EDTA. (**C**) Titration of the A98F hDUT mutant with EDTA shows no binding between the molecules. Titrations were carried out at pH 7.5 and at 293 K.

We used isothermal titration calorimetry (ITC) to obtain direct proof of EDTA binding to dUTPases. Figure 6 shows titrations of the human and mycobacterial dUTPases (panels A and B, respectively) with EDTA. The titration curves indicate exothermic binding between dUTPases and EDTA in the absence of Mg^2+^ ion. The thermodynamic parameters indicate strong binding with the K_d_ in the nanomolar range (199 nM for hDUT, and 78 nM for mtDUT) (Table 4). As our *in silico* results indicated that EDTA binds to the active site of dUTPases, we aimed to investigate this hypothesis *in vitro*. Thus, we performed the ITC measurement using an active site mutant of dUTPase. The previously described A98F hDUT mutant excludes dUTP from its active site as the phenyl ring of the F98 residue occupies the space of the uracil ring of dUTP [19]. As shown by the docking of EDTA to hDUT in Figure 4A, the binding site of EDTA overlaps with that of the uracil ring. Therefore, we expected that EDTA binding to the A98F hDUT mutant would be compromised if EDTA binding occurs in the dUTP binding pocket. The ITC titration of A98F hDUT with EDTA clearly shows that the active site mutant hDUT lost its EDTA binding capacity (Figure 6C). Thus, we concluded that EDTA binding to dUTPase primarily occurs in the nucleotide binding pocket.

As a control, we titrated the dUTPases with their practically non-hydrolysable substrate analogue dUPNPP in the absence of Mg^2+^ ion. Both hDUT and mtDUT exhibited exothermic dUPNPP binding (Supplementary Figure S1A,B, respectively). The obtained K_d_ value for the hDUT:dUPNPP complex is 44 µM. The same titration in the presence of Mg^2+^ has previously been done yielding K_d_ values between 2–7 µM [7,18,51]. Taking into consideration that Mg^2+^ supports a more favorable charge distribution within the substrate-bound active site [52], the obtained one order of magnitude increase in the K_d_ values without Mg^2+^ is reasonable.

**Figure 7:**
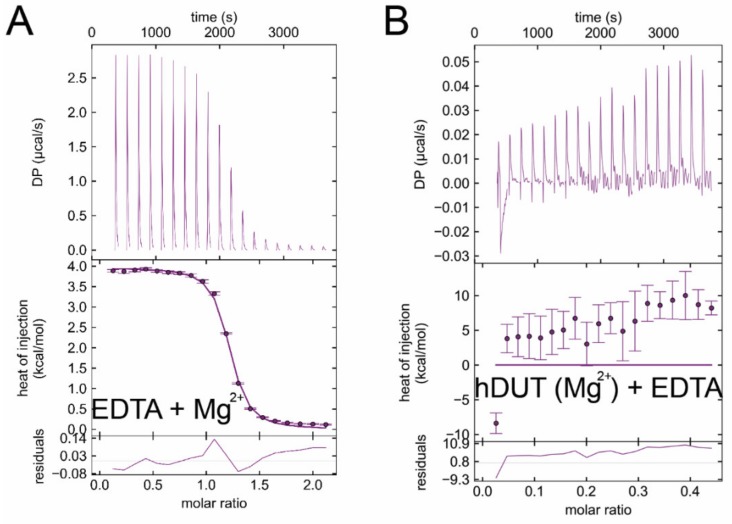
Control ITC measurements. (**A**) ITC titration of EDTA with MgCl_2_. (**B**) ITC titration of hDUT with additional 5 mM MgCl_2_ in the buffer with EDTA. Titrations were carried out at pH 7.5 and at 293 K.

We also performed EDTA titrations with Mg^2+^ as a control (Figure 7A). Mg^2+^ binding to EDTA proved to be an endothermic reaction well separable from EDTA binding to dUTPases. The dissociation constant of the complex is around 3 µM confirming literature data [58]. We performed a further control measurement, where we added extra Mg^2+^ into the buffer of hDUT and then titrated with EDTA (Figure 7B). Here we observed a mixed binding curve, suggesting that the exothermic binding of EDTA to the enzyme and the endothermic binding of EDTA to Mg^2+^ take place at the same time. As this curve is much different from the EDTA-hDUT or EDTA-mtDUT binding curves (Figure 6A,B), we could rule out the possibility of measuring the binding of potential residual Mg^2+^.

### 3.7. EDTA Binds to Taq Polymerase and MutT, Two Additional dNTP Processing Enzymes

**Figure 8:**
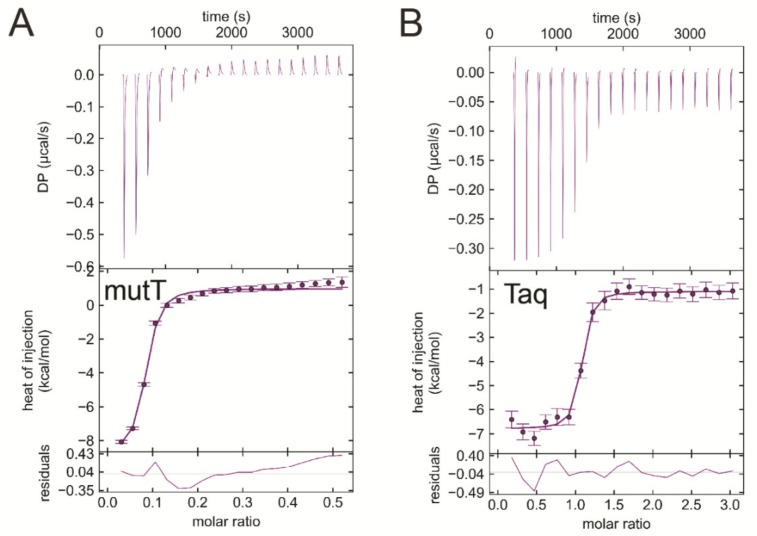
ITC titration of various dNTP hydrolyzing proteins with EDTA (**A**) EcMutT titration with EDTA. (**B**) *Thermus aquaticus* DNA polymerase titration with EDTA represents strong binding between the reactants. Titrations were carried out at pH 7.5 and at 293 K.

As EDTA occupied the dNTP binding site in both dUTPases investigated, we hypothesized that EDTA could bind other dNTP processing enzymes, as well. We selected two more enzymes, the *Escherichia coli* MutT (EcMutT) exhibiting 8-oxo-dGTPase activity [17] and the *Thermus aquaticus* DNA polymerase (Taq polymerase) [59] to test their ability to bind EDTA. Figure 8A shows the ITC curve of EcMutT and EDTA binding in the absence of Mg^2+^. Interestingly, the interaction of EDTA with EcMutT shows similar characteristics to that with dUTPases (Table 4). It is worth mentioning that EcMutT is a promiscuous enzyme which hydrolyses other dNTPs including dGTP, dCTP, dTTP, and dUTP beside 8-oxo-dGTP [60]. The Taq polymerase exhibits the strongest measured exothermic binding to EDTA in the absence of Mg^2+^ (K_d_ = 47 nM, Figure 8B, Table 4). This enzyme promiscuously processes all dNTPs.

We also tested enzymes that either have an ATP binding site (skeletal myosin S1) or have no nucleotide binding site at all (uracil glycosylase inhibitor and phenylalanine ammonia-lyase). As Supplementary Figure S2 shows, these proteins not having a dNTP binding pocket do not interact with EDTA in the ITC measurements.

## 4. Discussion

The controversy in the literature about dUTPase activity in the absence of metal(II) ions brought our attention to a putative, novel effect of EDTA on hydrolytic activity. We show, in effect, that EDTA competes with dUTP for the active site. EDTA is commonly used in biochemical experiments to establish metal-free conditions. However, EDTA has previously been reported to exhibit inhibitory effect on various types of enzymes via partially known (competitive inhibition of human liver arginase [61], electron donor in the reactions catalyzed by veratryl alcohol oxidase [62], and horseradish peroxidase [63,64]) or unknown mechanisms (prenyltransferase MaPT [65], peptidase activity of the angiotensin-converting enzyme [66]). Furthermore, a series of ethylenediamine-based farnesyltransferase inhibitors has been developed and evaluated as antimalarial and anticancer agents [67,68]. Nevertheless, the multifactorial effect of EDTA on enzymes has not penetrated the scientific community. Now that we show potent interaction with enzymes playing key cellular roles including a DNA polymerase and DNA repair enzymes, the view of EDTA being an inert biochemical reagent will hopefully be shifted.

**Figure 9:**
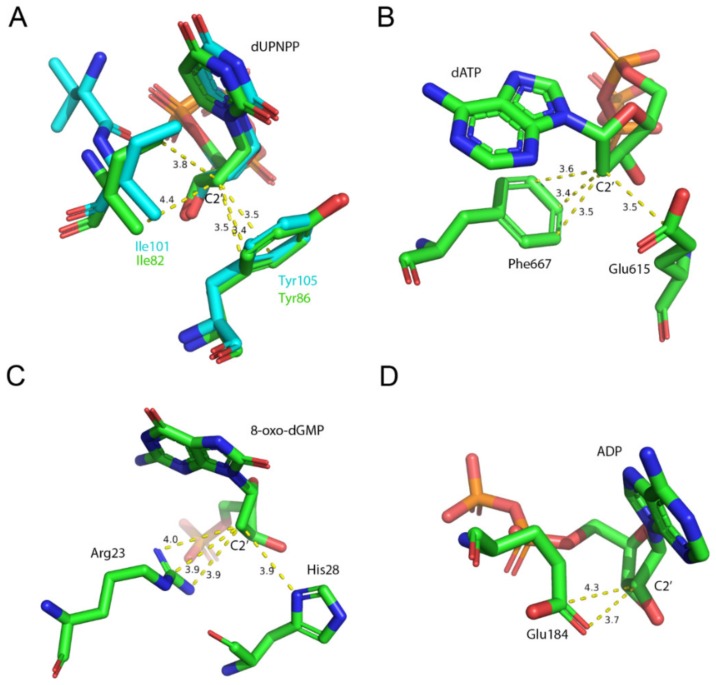
Molecular environment of the C2′ atom of the nucleotide within the active sites of the investigated (d)NTP hydrolyzing enzymes. Residues within 4 Å of C2′ are shown. (**A**) mtDUT (green, PDB ID: 2PY4 [20]) aligned with hDUT (cyan, PDB ID: 3EHW) both in complex with the non-hydrolysable dUTP analogue, dUPNPP. The conserved Ile and Tyr residues exclude a potential hydroxyl group on 2C’ atom. (**B**) The C2′ atom of the nucleotide within the Taq polymerse (PDB ID: 6Q4V [70]) fits in a sterically restricted environment. (**C**) *E. coli* MutT in complex with the hydrolysis product 8-oxo-dGMP (PDB ID: 3A6T [71]). A potential hydroxyl group on C2′ atom is similarly excluded as in the previous two enzymes. (**D**) Squid myosin S1 in complex with ADP (PDB ID: 3I5F [72]). The C2′ atom is sterically restricted from only one direction. The opposite side of the sugar plane faces the solvent.

EDTA inhibited the Mg^2+^-free dUTP hydrolysis activity of both dUTPases with similar mechanism but to a different degree (cf. Table 1). This observation is consistent with the results of the docking simulations and of the ITC measurements. We found that the inhibition comprises of a competitive and a non-competitive element in both dUTPases. The ITC experiments and the docking simulations showed that EDTA binds to the active site of dUTPase directly competing with the physiological substrate dUTP (Figure 4, Figure 5 and Figure 6). EDTA assumes somewhat different conformations though in the two different active sites despite the fact that the conformations of the dUTP ligand in the two structures are identical. The analysis of the interaction network within the EDTA bound active sites of hDUT and mtDUT also shows some differences (Table 3, Figure 5). However, the fact that EDTA exhibited strong interaction with four different dNTP hydrolyzing enzymes and no interaction with an ATPase calls our attention to enzyme residues that are responsible for the specificity of the nucleotide sugar (i.e., ribose (NTP) or 2′ deoxyribose (dNTP)). Taking a closer look at the protein environment of the C2′ atom of the bound nucleotide in all five of these enzymes (Figure 9), it is visible that the dNTP-specific enzymes (i.e., dUTPases, Taq, and MutT) sterically ensure the exclusion of the 2′OH while the NTP-specific enzyme, myosin does not. The residues shown in Figure 9 are responsible for the discrimination of the nucleotide sugars and are conserved in dUTPases [6] and in DNA polymerase I enzymes [69] and at least partially conserved in MutT (Figure S3). In the two dUTPases investigated, the same residues are involved in the interaction with EDTA (Figure 5, Ile101/82 and Tyr105/86). 

The striking results showing that EDTA occupies the active site in a manner similar to the nucleotide ligand urged us to obtain EDTA complexed dUTPase crystal structures. We put much effort in crystallization trials and collected several data sets from co-crystallized and soaked crystals as well. However, we could only observe potential molecular details of the acetate groups of EDTA within the active site of the mtDUT. After several trials, we concluded that EDTA, especially the ethylenediamine moiety might be too flexible to be observed in the crystal phase.

The interaction of EDTA with Mg^2+^ is endothermic while the interaction of EDTA with proteins proved to be exothermic. Thus, the two processes can be clearly separated from each other and any residual Mg^2+^ did not disturb the evaluation of EDTA binding to the proteins. While the EDTA:Mg^2+^ interaction is clearly driven by entropy predominantly due to the metal ion dehydration [73], EDTA binding to the proteins is more enthalpy-driven (Table 4). The relatively small entropy and larger enthalpy contribution to EDTA binding to the enzyme active sites is in accordance with our hypothesis that EDTA remains highly flexible within the active site while engaging in a significant number of secondary interactions with active site residues (Figure 5). Interestingly, the K_d_ vaIues of the enzyme:EDTA complexes are in the submicromolar range, 1–3 orders of magnitude lower than those of the Mg^2+^:EDTA or the enzyme:substrate complexes (Table 4) even in the presence of Mg^2+^ (K_d_ of the (Mg^2+^dNTP):enzyme complex being in the tens of micromolar range in Taq [59] and MutT [17], and in the low micromolar range in dUTPases [18,51]). 

The blind docking simulations suggested that EDTA may also bind to the surface of the enzyme potentially contributing the observed non-competitive type inhibition. The most populated EDTA binding surface areas differ in hDUT and mtDUT (Figure 3). EDTA seems to preferably bind to one of the entrances of the polar central channel of hDUT (Figure 3 A–B) while it only weakly populates the corresponding apolar area in mtDUT in the docking runs (Figure 3 C–D). Considering the enzymatic mechanism, EDTA binding to the outer central channel may not have great or any effect on the enzymatic activity as this part have not shown significant conformational changes in previous studies [55,74]. On the other hand, EDTA may perturb the interaction of the C-terminal arm with the protein core as this contact surface is the first and second most populated EDTA binding site in mtDUT and hDUT, respectively, in the docking simulations (Figure 3). The intact C-terminal arm–protein core interaction is necessary for the hydrolysis to occur as this interaction provides the shielding of the active site from the solvent [52,57,74]. The differences in the predicted EDTA-protein interactions, especially at the C-terminal arm can explain the observed differences in EDTA-inhibited dUTPase activities between hDUT and mtDUT. Using wild type and active site mutant dUTPases, we have conclusively identified the binding site to be the active site in the ITC measurements (Figure 6) and could not differentiate additional binding sites. The rather non-specific binding predicted on the molecular surface is not expected to be accompanied by a large thermal effect and therefore, the docking and the ITC results do not challenge each other.

The presented results call attention to the possibility that EDTA may be a binding partner/inhibitor of many more dNTP hydrolyzing enzymes. In addition, previous studies have shown that removal of EDTA from buffers might not be straightforward. EDTA is often retained in the dialysis bag even though the pore size is over hundred times larger than the molecule itself and buffer changing does not improve removal efficiency either [75]. Moreover, EDTA was reported to bind to both strong and weak anion exchange resins and to be only eluted at 240 mM NaCl, leading to 10-200 fold enrichment of EDTA in the elute compared to the initial solution [76,77]. Therefore, the common unsuspecting use of EDTA as a metal chelator during enzyme preparation procedures may introduce unwanted effects and misinterpretation of enzyme activity results. 

## 5. Conclusions

In conclusion, we found that EDTA is able to occupy the conserved dNTP binding sites in two dUTPases and potently binds to Taq polymerase and MutT, as well, while not interacting with the myosin ATPase. Our results together with the cited literature call attention to the fact that EDTA has multifactorial effects on several enzymes and therefore, the rethinking of the use of EDTA in enzymatic experiments is necessary. Considering that the chemical synthesis of substituted ethylenediamines is relatively straightforward, we propose that ethylenediamine-based inhibitors could be developed against DNA polymerases, various DNA repair and other nucleotide hydrolyzing enzymes for use in molecular biology experiments.

## Figures and Tables

**Table 1:** Kinetic parameters of hDUT and mtDUT in the presence of Mg^2+^ or EDTA or in the absence of both.

hDUT			mtDUT		
	k_cat_/s^−1^	K_M/_µM		k_cat_/s^−1^	K_M/_µM
5 mM Mg^2+^, no EDTA	5.27 ± 0.24	2.26 ± 0.33	5 mM Mg^2+^, no EDTA	2.39 ± 0.16	1.72 ± 0.51
No Mg^2+^, no EDTA	3.71 ± 0.12	2.49 ± 0.37	No Mg^2+^, no EDTA	0.79 ± 0.01	2.70 ± 0.18
30 µM EDTA, no Mg^2+^	3.30 ± 0.26	6.72 ± 1.69	300 µM EDTA, no Mg^2+^	0.71 ± 0.01	10.04 ± 0.50
100 µM EDTA, no Mg^2+^	0.24 ± 0.03	6.16 ± 5.34			

**Table 2:** Melting temperatures of hDUT obtained by thermofluor assay.

	T_m_ (5 mM Mg^2+^)/°C	T_m_ (No Mg^2+,^ no EDTA)/°C	T_m_ (100 µM EDTA)/℃
apo	59.5 ± 0.3	58.6 ± 0.1	58.5 ± 0.1
dUPNPP	63.9 ± 0.3	59.1 ± 0.2	58.3 ± 0.1
dUMP	62.3 ± 0.1	59.7 ± 0.1	58.7 ± 0.1

**Table 3:** H-bonding interaction points between dUTPase enzymes and EDTA or dUTP according to the active site dockings.

hDUT		mtDUT	
EDTA	dUTP	EDTA	dUTP
Arg85 (II)	Arg85 (II)	Arg64 (II)	Arg64 (II)
Ser86 (II)			
Gly87 (II)	Gly87 (II)	Gly66 (II)	Gly66 (II)
Gly99 (III)	Gly99 (III)		
		Thr81 (III)	
Asp102 (III)	Asp102 (III)	Asp83 (III)	Asp83 (III)
	Gly110 (III)		Lys91 (III)
	Gln131 (IV)	Gln113 (IV)	Gln113 (IV)
Arg153 (V)	Arg153 (V)	Arg140 (V)	Arg140 (V)
			His145 (V)
Ser160 (V)	Ser160 (V)	Ser147 (V)	Ser147 (V)
Thr161 (V)	Thr161 (V)	Ser148 (V)	Ser148 (V)

Roman numerals in parenthesis represent the adequate conserved motifs, rows represent identical positions in the 3D structure [6].

**Table 4:** Thermodynamic parameters yielded by the analysis of the ITC measurements. K_d_ values are apparent dissociation constants at pH 7.5 and at 293 K. Values shown are averages and standard deviations.

Titrand	Titrant	ΔH/kcal/mol	−TΔS/kcal/mol	ΔG/kcal/mol	K_d/_μM
hDUT	EDTA	−11.2 ± 0.4	2.2 ± 0.4	−9.0 ± 0.0	0.20 ± 0.009
hDUT	dUPNPP	−17.4 ± 3.6	11.5 ± 3.7	−5.9 ± 0.2	43.9 ± 14.6
mtDUT	EDTA	−4.2 ± 1.0	−5.3 ± 1.2	−9.6 ± 0.2	0.078 ± 0.023
mtDUT	dUPNPP	−4.5 ± 0.2	−2.1 ± 0.4	−6.6 ± 0.6	13.9 ± 11.7
EDTA	Mg^2+^	4.4 ± 0.3	−12.5 ± 0.6	−8.0 ± 0.9	2.53 ± 0.18
EcMutT	EDTA	−9.7 ± 0.02	1.3 ± 0.1	−8.4 ± 0.1	0.55 ± 0.066
Taq polymerase	EDTA	-6.3 ± 0.7	−3.5 ± 0.7	−9.8 ± 0.0	0.047 ± 0.001

## Supplementary Materials

The following are available online at https://www.mdpi.com/2218-273X/9/10/621/s1, Figure S1: ITC titrations with dUTPases and dUPNPP confirm the functionality of the enzyme, Figure S2: ITC titrations show the lack of binding between EDTA and *Staphylococcus aureus* uracil glycosylase inhibitor, *Petroselinum crispum* phenylalanine ammonia-lyase and rabbit myosin subfragment 1, Figure S3: The alignment of MutT protein sequences indicate that the residues involved in 2′ deoxyribose exclusion from the active site are conserved amongst the above species.
