# Supplementary material for: Beyond Chelation: EDTA Tightly Binds Taq DNA Polymerase, MutT and dUTPase and Directly Inhibits dNTPase Activity
Source: Biomolecules. 2019 Oct 17;9(10):621. doi: 10.3390/biom9100621 (PMC6843921; doi:10.3390/biom9100621)
Supplement: Supplementary file 1 [file biomolecules-09-00621-s001.pdf]

Supplementary data to

Beyond chelation: EDTA tightly binds Taq DNA polymerase, MutT and dUTPase and directly inhibits dNTPase activity

Anna Lopata[1,2], Balázs Jójárt[3], Éva V. Surányi[1,2], Enikő Takács[1], László Bezúr[4], Ibolya Leveles[1,2], Ábris Á. Bendes[1], Béla Viskolcz[5], Beáta G. Vértessy[1,2] and Judit Tóth[1,*]

[1] Institute of Enzymology, Research Centre for Natural Sciences, Hungarian Academy of Sciences, Budapest, 1113, Hungary
[2] Department of Applied Biotechnology, Budapest University of Technology and Economics, Budapest, 1111, Hungary
[3] Institute of Food Engineering, Faculty of Engineering, University of Szeged, Szeged, 6724, Hungary
[4] Department of Inorganic and Analytical Chemistry, Budapest University of Technology and Economics, Budapest, 1111, Hungary
[5] Institute of Chemistry, University of Miskolc, Miskolc, 3515, Hungary

* To whom correspondence should be addressed. toth.judit@ttk.mta.hu, Tel: +36 1 3826793

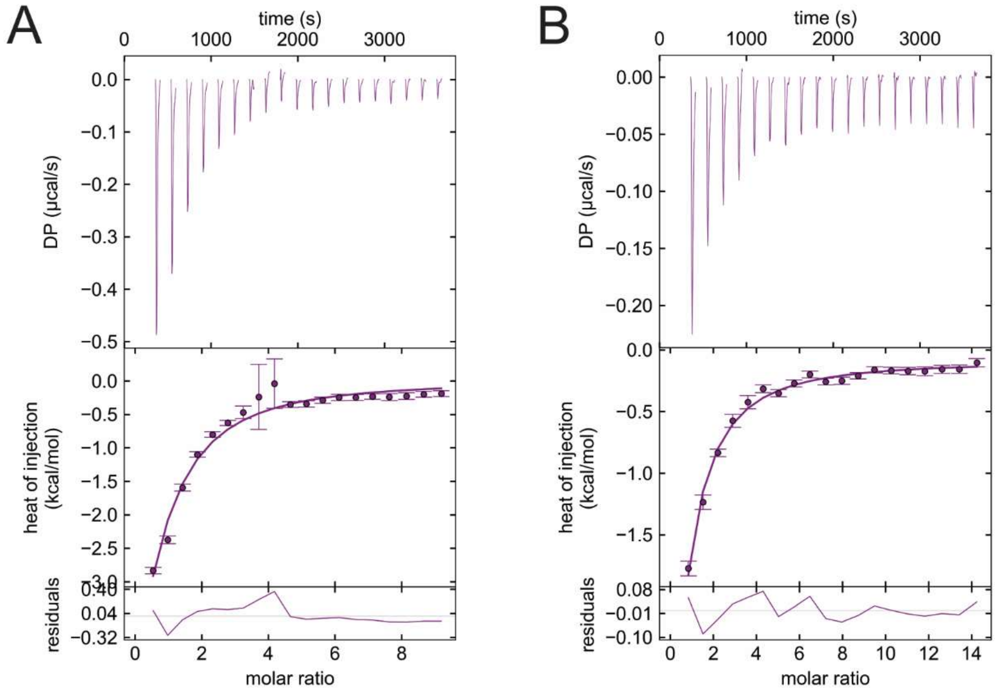

Figure S1 ITC titrations with dUTPases and dUPNPP confirm the functionality of the enzyme. A) hDUT and dUPNPP titration B) mtDUT and dUPNPP titration represent the binding between the components. Parameters of the fitting are the following: A) ΔH = –19.9 kcal/mol, $-T\Delta S$ = 14.1 kcal/mol, ΔG = –5.7 kcal/mol, K_d = 54 μM, n = 0.41; B) ΔH = –4.4 kcal/mol,

$-T\Delta S$ = –1.8 kcal/mol, ΔG = –6.2 kcal/mol, K_d = 22 μM, n = 1.

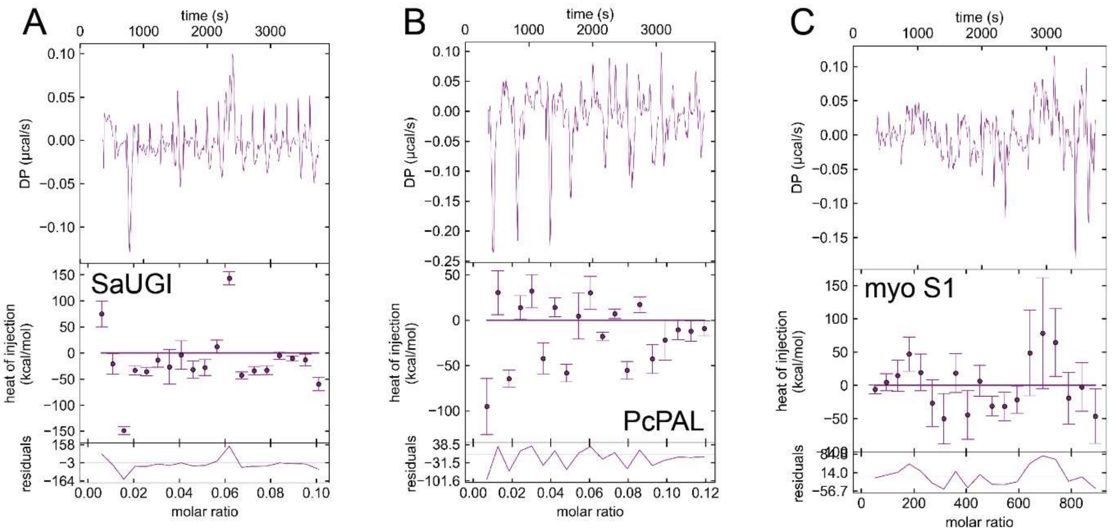

Figure S2 ITC titrations show the lack of binding between EDTA and A) *Staphylococcus aureus* uracil glycosylase inhibitor (SaUGI), B) *Petroselinum crispum* phenylalanine ammonia-lyase (PcPAL) and C) rabbit myosin subfragment 1 (myo S1).

```
coli            -------MKKLQIAVGIIRNENNEIFITRRAADAH--MANKLEFPGGKIEMGETPEQAVV
archaea         MADQAHPVREVHVAVGVVQRRGR-VLIARRPDHAH--QGGLLEFPGGKVEPGETVQQALI
m.tub           --------MLNQIVVAGAIVRGCTVLVAQRVRPPE--LAGRWELPGGKVAAGETERAALA
m.smeg          --------MTKQIVVAGALISRGTLLVAQRDRPAE--LAGLWELPGGKVTPGESDADALA
streptococcus   ---------MPQLATICYIDNGKELLMLHRNKKPNDVHEGKWIGVGGKLERGETPQECAA
                           ::..         ::: :*   ..    .     ***:  **:   .

coli            RELQEEVG--ITPQHFSLFEKLEYEFPDRHITLWFWLVERWEGEPWGKEGQPGEWMSLVG
archaea         RELAEETGLRVSPDALEPLIGIRHDYGDKRVFLDVWRTGQAEGEPEGREGQAVAWLAPEA
m.tub           RELAEELGLEVADLAVGDRVGDDIALNG-TTTLRAYRVHLLGGEPRARDHRALCWVTAAE
m.smeg          RELREELG---VDVAVGERLGADVALND-AMTLRAYRVTLRSGSPHPHDHRALRWVGADE
streptococcus   REILEETG-LKAKPVLKGVITFPEFTPDLDWYTYVFKVTEFEGDLIDCNEGTLEWVPYDE
                **: ** *       .           .       : .    *.    :  .  *:

coli            LNADDFPPANEPVIAKLKRL----------------------------------------
archaea         LRDEDFPAANRPIIRALRLPQTLAITGHVRSVGDGLAALTASLDRTPVSALVLRAPALDD
m.tub           LHDVDWVPADRGWIADLARTLNGSAADVHRRC----------------------------
m.smeg          IDGIAWVPADRAWVPDLVAALSGR------------------------------------
streptococcus   VLSKPTWEGDHTFVEWLLEDKPFFSAKFVYDGDKLLDTQVDFYE----------------
                :       .:.  :  *
```

Figure S3 The alignment of MutT protein sequences indicate that the residues involved in 2' deoxyribose exclusion from the active site (green) are conserved amongst the above species. Arg23 can be found in *Escherichia coli*, *Streptococcus pneumoniae*, *Mycobacterium tuberculosis*, *Mycobacterium smegmatis* and in *Euryarchaeota archaeon*. His28 is additionally conserved in *Escherichia coli* and in *Euryarchaeota archaeon*. However no homologue residues were found in MutT proteins from *Bacillus subtilis*, *Haemophilus influenzae*, *Arabidopsis thaliana* and *Homo sapiens*. This region is not part of the conserved motif identified by Koonin 1993 NAR.

Aligned proteins:

>sp|P08337|MUTT_ECOLI 8-oxo-dGTP diphosphatase OS=Escherichia coli (strain K12) OX=83333 GN=mutT PE=1 SV=1

>sp|P41354|MUTX_STRPN 8-oxo-dGTP diphosphatase OS=Streptococcus pneumoniae serotype 4 (strain ATCC BAA-334 / TIGR4) OX=170187 GN=mutX PE=1 SV=2

>tr|A0A2E4GCL2|A0A2E4GCL2_9EURY DNA mismatch repair protein MutT OS=Euryarchaeota archaeon OX=2026739 GN=CL958_03010 PE=4 SV=1

>sp|P9WIY1|MUTT2_MYCTU Putative 8-oxo-dGTP diphosphatase 2 OS=Mycobacterium tuberculosis (strain ATCC 25618 / H37Rv) OX=83332 GN=mutT2 PE=1 SV=1

>tr|A0A0D6IWC3|A0A0D6IWC3_MYCSM Mutator protein MutT2/NUDIX hydrolase OS=Mycobacterium smegmatis OX=1772 GN=mutT2 PE=3 SV=1
